# Deep Residual Network for Smartwatch-Based User Identification through Complex Hand Movements

**DOI:** 10.3390/s22083094

**Published:** 2022-04-18

**Authors:** Sakorn Mekruksavanich, Anuchit Jitpattanakul

**Affiliations:** 1 Department of Computer Engineering, School of Information and Communication Technology, University of Phayao, Phayao 56000, Thailand; sakorn.me@up.ac.th; 2 Department of Mathematics, Faculty of Applied Science, King Mongkut’s University of Technology North Bangkok, Bangkok 10800, Thailand; 3 Intelligent and Nonlinear Dynamic Innovations Research Center, Science and Technology Research Institute, King Mongkut’s University of Technology North Bangkok, Bangkok 10800, Thailand

**Keywords:** user identification, deep learning, smartwatch sensor, residual network, squeeze-and-excitation block

## Abstract

Wearable technology has advanced significantly and is now used in various entertainment and business contexts. Authentication methods could be trustworthy, transparent, and non-intrusive to guarantee that users can engage in online communications without consequences. An authentication system on a security framework starts with a process for identifying the user to ensure that the user is permitted. Establishing and verifying an individual’s appearance usually requires a lot of effort. Recent years have seen an increase in the usage of activity-based user identification systems to identify individuals. Despite this, there has not been much research into how complex hand movements can be used to determine the identity of an individual. This research used a one-dimensional residual network with squeeze-and-excitation (SE) configurations called the 1D-ResNet-SE model to investigate hand movements and user identification. According to the findings, the SE modules have enhanced the one-dimensional residual network’s identification ability. As a deep learning model, the proposed methodology is capable of effectively identifying features from the input smartwatch sensor and could be utilized as an end-to-end model to clarify the modeling process. The 1D-ResNet-SE identification model is superior to the other models. Hand movement assessment based on deep learning is an effective technique to identify smartwatch users.

## 1. Introduction

Annually, the quantities of information produced by wearable devices linked to the Internet increase [1]. Smartphones play a significant part among these gadgets because of their increasing functionality and consumer acceptability. As a result, the safety and security of this equipment are the top priority throughout the design phase. Biometrics can be employed in several of the newest strategies for controlling illegal access to mobile devices. An individual’s observable characteristics and behaviors are examined and measured to recognize or identify that person.

User authentication is an excellent way to protect personal information. The design of the authentication mechanism must take into account the fact that the aim of authenticating is to validate the user’s information [2]. In preventing identity theft, many new solutions have been introduced in recent years. Identifying the user and providing a pleasant user experience are the primary goals of these detection techniques, but there are still several obstacles to overcome. Digital identity is increasingly built on usernames and passwords [3], making it vulnerable to theft, hacking, and fraud. Cryptographic algorithm-based digital signatures are another common choice [4]. A highly capable computer system is needed to produce digital signatures; therefore, devices with fewer resources have difficulties establishing this identification. Hardware-based PUF (Physical Unclonable Function) has recently emerged to identify individuals, and several authentication methods have been constructed on this basis [5]. There are certain drawbacks to PUF, however, including the need for additional equipment. A hardware-based identification solution is implemented via tokens and access cards [6].

A biometrics-based identification approach is the next advance in identifying and verifying individuals [7]. Due to obvious reasons, individuals are regarded more efficiently than the previously listed digital IDs. Because they are a part of ourselves, biometrics are easy to utilize. Compared to more conventional verification and identification methods, including credentials, PINs, and tokens, biometrics are almost impossible to lose or steal [8]. Secondly, since each person’s biometrics are distinctive, they are complicated to reproduce. It is also easy to verify the properties of biometric IDs [9]. Modern computer systems have several physiological biometrically based identifiers. Several products employ face detection to verify that the individual is who they indicate they are. When it comes to biometrics, the fingerprint is the most often utilized [10]. There are a number of common biometric signatures, including ECG/EEG characteristics [11], iris recognition [12], and palm vein variations [13]. All of these options need specialized technology to collect biometric data. This could be prohibitively costly, time consuming, and intrusive to the participant. A further drawback of these physiologically based approaches is that they are vulnerable to emulation. Fraudulent activity includes voice impersonation, iris-copying lenses, and concealment, and these are but a few examples.

Many emerging biometric identification alternatives are low cost, better suited than classic biometrics, or could be used in conjunction with more classical biometrics like multi-factor authentication to enhance security and usability. On the other hand, some biometric authentication methods demand human engagement, which might be difficult for the end-user. A few alternatives include entering the password, unlocking the smartphone via face recognition, or tapping the fingerprint reader. In continuous authentication, the user is required to authenticate many times [14]. This is more challenging for the user. Since biometric characteristics are collected indirectly when the user interacts with the device, movement sensor-based identification approaches such as wearable sensor-based gait recognition [15], contact gesture-based recognition, keystroke-based recognition, etc., could tackle this issue. Compared to standard vision-based movement identification, these methods are more private [16] and use less energy.

The advancement of science and technology has influenced the techniques of biometric identification. Fingerprinting, facial detection, retinal scanning, palm geometry, and voice recognition are some of the most well known techniques, but there are many more. Meanwhile, less invasive biometric variations are making their way into widespread utilization. Identifying persons based on the features of their activity is an example of this. There are both pros and cons to this approach, which indicate how individuals perform their daily routines. Since the person needs to carry the equipment (which will generally be a smartphone) or be captured to perform recognition by computer vision, its key benefits are that it enables automated, regular, and non-intrusive recognition. Biometric techniques that are less accurate than fingerprints might be seen as a drawback. Continuous and periodic identification could help with this. For gait-based person recognition systems, performance can be improved if the data samples are created by a broad range of user actions, ensuring that a valid classification is achieved in the least amount of time feasible.

In many homes and workplaces, smartphones have become an integral part of our daily lives and are routinely used to access cloud-based security apparatuses. Smartwatches offer an interesting environment for authentic identity verification through cloud-based solutions like Internet banking if a smartphone is easily stolen or cobbled. When using cloud-based or other data sources to connect mission-critical Internet services, it is vital to identify the genuine user who is doing so reliably. Automated and non-bypassable identification is required.

In the recent decade, learning techniques, including machine learning (ML), have been employed to achieve good outcomes with biometric-based user identification. Within controlled circumstances, machine learning techniques such as K-nearest neighbors, Support Vector Machine (SVM), Decision Tree (DT), and Random Forest (RF), among others, have been established to deliver satisfactory results [17,18,19]. The accuracy of these standard machine learning models is highly dependent on the strategy of human-manually extracted and selected features.

Nowadays, deep learning (DL) algorithms have succeeded in user identification studies. One of the most significant components of deep learning is its ability to automatically identify and classify features with increased accuracy, influencing user identification studies [20,21,22]. Deep neural networks can learn discriminative characteristics from raw data automatically, and they have revealed tremendous promise for evaluating diverse data and have a high capacity for generalization. Numerous primary and sophisticated deep learning models have been proposed to capitalize on deep learning approaches by compensating for the drawbacks of traditional machine learning while leveraging the multiple levels of characteristics available in various hierarchies. A hierarchy of layers is used in machine learning techniques to handle low- and high-level features, along with linear and nonlinear feature conversions at different levels, contributing to learning and optimizing features. To this end, deep learning models such as Recurrent Neural Networks (RNN), Convolutional Neural Networks (CNN), and Long Short-Term Memory (LSTM) are utilized to overcome the limitations of conventional machine learning algorithms that relied on manual feature selection, where an error in feature selection could have negative consequences for the applications at hand. As a result, deep learning networks have found practical applications in identification tasks and are often employed in activity recognition studies for feature extraction. One disadvantage of the deep learning approach, mainly when complex DL architectures are used, is the higher expense of processing the massive number of accessible datasets. Nevertheless, the cost is justified since an identification approach relies on the accuracy of the classification consequences of the deep learning model.

While enhancing the level of a CNN could extract more abstract features and improve effectiveness, it may also result in degraded performance [23]. To address this issue, He et al. [24] suggested a residual network (ResNet) for image identification, which has been integrated into the investigation of human behavior. For instance, Li et al. [25] used the 1D-ResNet model to extract spatial features from multidimensional inertial sensor inputs and bidirectional LSTM. This approach can achieve improved performance with fewer parameters. Moreover, Ronald et al. [26] established a link between wearable sensor vectors and individual motions using an improved deep learning model based on ResNet and Inception modules. The improved model suggested here shows exceptional arrangement in human activity recognition (HAR) implementations.

Using a squeeze-and-excitation (SE) module [27] is a technique for channel attention that could be contained in current CNNs to increase classification interpretation. The SE block operates as an embedding unit, improving the effectiveness of deep neural networks. The SE block is one of the popular improvements of the many CNN architectures because it is simply added without changing the shape of the existing model. For example, [28,29,30] concatenated the SE block into CNNs with varying convolutional layer levels. The findings indicate that CNNs with the SE block outperform simple CNNs with accuracy rates. Additionally, the efficiency of the SE block has been shown in the assessment of pork freshness using NIRS [31] and ECG-signal classification [32].

Inspired by the works mentioned earlier, this research combines the SE block with a 1D-ResNet to evaluate the SE block’s potential for user identification using sensor data from a smartwatch. The main difference between the proposed network and our previous works in [28,29,30] is that the proposed network uses a one-dimensional deep residual network with shortcut connections instead of a one-dimensional convolutional neural network. This work aims to investigate user identification using a smartwatch on the basis of complex hand movements. We use a residual network to extract more abstract spatial characteristics from CNN. Squeeze-and-excitation modules were also incorporated in the one-dimensional ResNet to improve recognition interpretation.

The following is an overview of the study’s most significant contributions:

- This article aims to investigate the possibility of the 1D-ResNet-SE for sensor-based user identification by analyzing complex hand movement signals captured by smartwatch sensors. We compared standard CNN-based deep learning models to RNN-based LSTM networks for sensor-based user identification using smartwatch sensor data to examine the algorithm’s effectiveness.
- We conducted comprehensive experiments using many smartwatch-based HAR datasets encompassing simple and complex hand movements to increase ecological validity. We observed the connection between hand motion patterns and the recognition of smartwatch owners using the 1D-ResNet-SE model. The SE blocks are combined with the residual network to increase the sensitivity to relevant features. Compared to CNN- and LSTM-based deep learning models, the model demonstrated here shower superior performance in user identification in complex hand movement situations.

The rest of this article is organized as follows: In the second section, we examine the latest research on sensor-based deep learning algorithms for user identification. Proposals for new techniques are introduced in Section 3. Experiments are described, and findings are shown in Section 4. Deep-learning algorithms used in the research to achieve effectiveness in identification are discussed in detail in Section 5. The last section, Section 6, focuses on the study’s limitations and suggests new opportunities for future research.

## 2. Related Works

Identifying users based on their activities has proved to be a challenging problem to solve. We have compiled a collection of resources connected to our study in this area.

### 2.1. Sensor-Based User Identification

Wearable sensors have been considered in recent years as part of sensor-based identification systems. For example, [33,34] proposed a mechanism for explicitly and continuously identifying the individual. However, there is no compelling evidence that the individual’s behavior has changed enough to warrant an apparent classification in most circumstances. Luca et al. [35] present a technique for explicitly determining the distance between pattern traces using the dynamic time warping mechanism. Most of the 22 unusual touch patterns shown by Sae-Bae et al. [36] include using all five fingers concurrently. They used k-nearest neighbors and support vector machines to categorize the 22 analytical characteristics from touch traces analyzed in the study [37].

As a result, each action is correlated with two essential characteristics: Time and space, according to the behavior-based model’s principle. For example, if you are looking to identify a user, you could look to activities such as those described by [38]. Multi-model continuous user identification was suggested by the works of [39]. Another distinctive architecture for ongoing user identification was proposed in [40] by leveraging historical smartphone records and positions.

To a certain degree, all the tasks mentioned above need additional details and a source of user identification. Casale et al. [41] provided a gait-based user identification over an inconspicuous biometric pattern to address these concerns. A four-layered structure made use of the geometric principle of a convex hull. For example, it only functions in specific locations which is a severe disadvantage. Wearable devices based on gait signals recorded using a three-dimensional accelerometer were employed in the studies of [8,42], where the accelerometer was simply attached to the individual’s waist at the rear. A three-fold technique for user identification based on data distribution statistics, correlation, and time-frequency characteristics was developed by [43]. At the same time, the people were deliberately requested to stroll at different velocities, such as slow, regular, or quick. The fundamental disadvantage of Mantyjarvi’s work is that only one person can walk at a time, and with relatively restricted variants.

There are many existing approaches that used gait-based systems which are summarized above and in Table 1. There is still scope for further improvement based on physical adjustments, carrying objects, orientation, placement, movement surface, psychosocial factors of a participant, stimulants, and other considerations. These limitations significantly hamper the gait-based system’s performance in real-world situations.

### 2.2. Deep Learning Approaches for User Identification

#### 2.2.1. Convolutional Neural Network

Several research studies on time series classification (TSC) have focused on deep learning and obtained notable results in recent years. CNN has been a prominent deep learning technique in TSC because of its capability to extract the connection between local organizations in the form of array information. Yang et al. [46] reveal one of the first applications of CNN in TSC. According to the researchers, a higher-level description of raw sensor data can be derived using CNN’s deep architecture. Additionally, combining feature learning and classification in a single model makes the learned features more discriminative. According to Ronao and Cho [47], a deep CNN with 1D convolutional processes outperforms conventional pattern recognition algorithms for movement categorization employing smartphone sensors. Jiang and colleagues [48] sent the sensor data into a two-dimensional neural network instead of utilizing a one-dimensional convolution to capture both temporal and spatial characteristics from the action patterns for the classification test. The two-stage CNN model [49] increases the classification performance of actions with complicated structures and limited training data.

TSC has subsequently benefited from the use of numerous cutting-edge CNN architectures that have been introduced in the machine vision area. A TSC model based on U-Net [50] was presented in [51] to conduct sampling point-level forecasting, thereby overcoming the multi-class issue. Mahmud et al. [52] use a residual block-based CNN to extract features and categorize behaviors from 1D time-series sensor data. TSC’s compact deep convolutional neural network is constructed by Tang et al. [53] using the Lego filter [54].

#### 2.2.2. Recurrent Neural Networks

Time series sensor data are commonly processed using recurrent neural networks (RNNs) because they store insights into the history of previous items in a sequence. Zeng et al. [55] presented a long short-term memory (LSTM) model based on continuous attention that emphasizes relevant sensor modalities and significant sections of the sensor data during TSC analysis. Barut et al. [56] constructed a multitask framework employing layered LSTM layers to classify and estimate activity intensity from raw sensor data. Rather than utilizing raw data, the bidirectional LSTM recurrent neural network in [57] is employed to feature data generated from principal component analysis (PCA) and discrete wavelet transform (DWT). Where there is a lack of label data, [58] recommends extracting features using spectrograms. Then the identification is carried out using an extended support vector machine (SVM). Fusing LSTM-RNN with handcrafted elements could improve the performance of a system, according to [59], where it was shown. Local feature-based LSTM networks suggested by Chen et al. [60] can encode temporal dependence and learn features from a high sampling rate of acceleration data.

#### 2.2.3. Hybrid Neural Networks

In recent years, considerable research has demonstrated that substantial TSC effectiveness could well be achieved by combining hybrid models derived from several kinds of deep learning approaches. GRUs (gated recurrent units) have been introduced in [61] to uncover sequential temporal relationships in complex activity recognition by using an inception module-based CNN [62]. In a sleep-wake detection system, Chen et al. [63] employed a 1D- CNN-LSTM model to capture feature information from lengthy acceleration sequences, then combined an attention mechanism with the handcrafted characteristics of heart rate variability data. In [64], a recurrent convolutional attention model was presented to cope with the imbalance of the labeled data in a semi-supervised manner. Small segments of window data are supplied into an LSTM layer for motion identification after a CNN is applied to the data. For the first time, an LSTM-CNN model was suggested by Xia et al. [65] in which a two-layer LSTM is applied topically to the raw sensor data before actually employing 2D convolutional layers. Deep learning and traditional pattern recognition approaches are successful in the research; however, further examination exposes several gaps and flaws. Rather than evaluating the connection between neighboring windows, most research has solely looked at the data from specific windows to make predictions about behavioral aspects. In multi-class classification applications, including face recognition, this technique could deliver great accuracy, but it can lack the characteristic of long-term reliance on sensor data. A method named MFAP, developed by Chen et al. [59], addresses this weakness by considering both the past and present a priori data. To maintain the assumption of independence between the observed values and the preceding ones, we consider the activity sequence a first-order Markov chain. This strategy, unfortunately, necessitates an additional manual job on the result of the deep neural network’s Softmax layer.

There has also been some research involved to apply and evaluate the principles under real-life scenarios; however, most past research employs clean datasets. The data of each action are gathered, interpreted, and preserved independently without taking transitions between movements into consideration. In reality, activities must be performed sequentially, and some, such as lying down and jogging, cannot be performed side by side without a transition. A hierarchical hybrid approach, known as HiHAR, has been proposed to overcome these issues. The process can determine local and global temporal dependence in window sequences using the hierarchical design.

### 2.3. Simple and Complex Human Activities

Human activities could well be classified into two categories according to [66,67,68,69]: Simple human activities (SHA) and complex human activities (CHA). As Shoaib et al. [70] observed, simple human activities are repeated, common movements that could be primarily determined using an accelerometer, such as strolling, running, sitting, and standing. Another issue is that behaviors that are not repeatable, such as smoking, eating, delivering a speech, or sipping coffee, cannot be clearly detected at smaller segmentation windows, in contrast to repetitive activities such as walking, running, or cycling. Human behaviors that are complex are less repetitive than those that are simple. Complex activities often need the use of the hands, such as smoking, eating, and drinking. Additional sensors, like a gyroscope, could be utilized to determine if CHA is present. Due to the difficulty of characterizing such actions with a single accelerometer, this research categorized stair-related movements as CHA.

Alo et al. [66] distinguished two types of human activities: Simple and complex. Walking, running, sitting, standing, and jogging are simple human activities that are quick human behaviors. On the other hand, complex human activities, such as smoking, eating, taking medicine, cooking, and writing, are composed of longer-duration operations. Peng et al. [67] divided human activities into simple ones (e.g., walking, jogging, or sitting) based on repetitive movements or a single body position, which does not genuinely describe everyday activities. On the other hand, complex activities are more challenging and are composed of many straightforward operations. Complex actions, such as eating breakfast, office working, or shopping usually require an extended period of time and have broad meanings. These are more accurate components of people’s everyday lives. According to Liu et al. [68], human activity is complicated. A complex activity is a collection of chronologically and productively related atomic engagements. In contrast, an atomic movement is a single unit-level action that cannot be further decomposed under practical comprehension. Rather than doing a single atomic operation, individuals frequently perform several activities in various ways, both sequentially and simultaneously. Chen et al. [69] distinguished two types of human activities: Simple and complex. SHA could be considered as a single repeated motion that a single accelerometer could recognize. CHA would rarely occur in repeatable form similar to simple activities and will usually include many simultaneous or overlapping actions that can be observed only via multimodal sensor data.

### 2.4. Available Sensor-Based Activity Datasets

Many sensor-based activity datasets are accessible to the public and could develop deep learning models.

All 51 individuals in the WISDM-HARB dataset [17] were recorded while participating in 18 activities of daily life. Each participant wore a smartwatch on their dominant wrist while completing the tasks to ensure accuracy. The research goal was to identify which integration form of accelerometer and gyroscope sensors achieved the best performance on both smartphones and smartwatches.

Smartwatch and smartphone data loggers are included in the UT-Smoke dataset [71,72] to collect various sensor data simultaneously. For three months, the participants in this study smoked for a total of 17 hours while strolling, standing, sitting, or speaking with others. Eleven people volunteered to take part in these events. According to our knowledge, this is the most significant dataset compared to other research of this type.

Annotated data from complicated hand-based movements recorded by smartwatches are utilized as a baseline for complex hand movement studies in the two datasets above [73,74,75]. UT-Complex [70], PAMAP2 [76], and OPPORTUNITY [77] are further sensor-based activity datasets. On the other hand, this research did not include data from an annotated smartwatch sensor for sophisticated hand-based tasks.

## 3. Proposed Methodology

**Figure 1:**
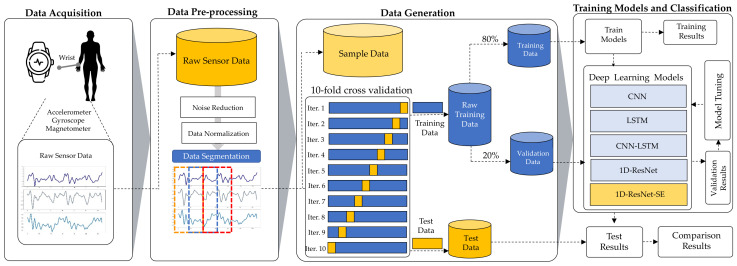
The proposed CHM-UserIden framework for smartwatch-based user identification used in this study.

An emphasis is placed in this part on the methods used for the training of the advanced learning model and the identification of individuals through wearable sensors and smartwatches which incorporate built-in sensors. In Figure 1, the proposed methodology for the CHM-UserIden framework is shown, which comprises data acquisition, pre-processing, training the model, and user identification. Each stage is explained in further detail as follows.

### 3.1. Data Acquisition

The benchmark datasets utilized to evaluate this research were the focus of this section. The assessment employed two public datasets (UT-Smoke and WISDM-HARB datasets). The inertial data from smartwatch sensors were included in the UT-Smoke and WISDM-HARB datasets. As a group of individuals engaged in everyday tasks, such as dining, having a drink, smoking, and so on, the data in each dataset were gathered. Data from accelerometer, gyroscope, and magnetometer-equipped smartwatch sensors were used to produce these datasets.

To investigate user identification through a smartwatch, we classified human activities using the SC${}^{2}$ representational taxonomy [68]. This division of human activities into simple and complex ones was based on their chronological interconnections.

- A simple activity cannot be subdivided further at the atomic scale. For instance, walking, running, and ascending are all considered simple activities owing to their inability to be coupled with other activities.
- A complex activity is a high-level activity formed via the sequencing or overlapping of atomic-level activities. For example, the representation ”smoking while strolling” incorporates the two atomic actions of ”strolling” and ”smoking”.

Characteristics of both activity-based datasets are described in Table 2.

#### 3.1.1. UT-Smoke Dataset

The UT-Smoke dataset, which was previously provided in [71,72], is used in this study as a public complex hand-based activity dataset. Over three months, 11 volunteers (two female and nine male) aged 20–45 were tracked using a smartwatch application. The program records data from a smartwatch and a smartphone’s triaxial accelerometer and gyroscope, as well as a timestamp. 50 Hz is the sampling rate for all data. The activities included smoking while standing (SmokeSD), smoking while sitting (SmokeST), smoking while walking (SmokeW), smoking in a group chat (SmokeG), drinking while standing (DrinkSD), drinking while sitting (DrinkST), dining, standing, sitting, and walking (Walk). Identifying smoking and behaviors comparable to smoking are the primary goals of this dataset. Every individual takes part in every activity except for SmokeG and SmokeW, which are carried out by a combined total of eight and three individuals, respectively.

#### 3.1.2. WISDM-HARB Dataset

Fifty-one people were recruited to participate in the WISDM-HARB dataset [17] and complete various everyday tasks, including easy and sophisticated studies using smartphones and smartphone sensors. The subjects performed these tasks for three minutes. The accelerometer and gyroscope sensors recorded data at 20 Hz. Individuals aged 19 to 48 volunteered to participate in the study, which collected sensor data.

### 3.2. Data Pre-Processing

Due to the participants’ lively motions throughout the data collection, raw sensor data contained measurement noise and other unanticipated noise. Signals with a lot of noise distort the data they convey. As a result, it was critical to limit the impact of noise on signal processing so that useful information could be retrieved from the signal [42,78]. Mean, low-pass, and Wavelet filtering are some of the most frequently used techniques for filtration. Using a 3rd order Butterworth filter, we de-noised all three dimensions of accelerometers, gyroscopes, and magnetometers using the 20 Hz cutoff frequency. At this pace, 99.9% of body movements are captured, making it ideal for the recording of motion [79].

It was necessary to alter the sensor data once it had been cleansed of unwanted noise. Each data point was transformed using a Min–Max normalization approach, which projects its values into the range [0, 1]. Having a way to balance the impacts of different dimensions might be beneficial for the learning processes. Normalized data from all sensors are split into equal-sized sections for model training using fixed-size sliding windows in the data segmentation stage of the process. To construct sensory data streams with a length, we employed a sliding window with a duration of 10 s in this study as suggested by [17]. The 10-s window is utilized for user identification because it is long enough to record crucial features of a person’s activities, such as numerous repeats of fundamental motions such as walking and stair ascending, and it enables faster biometric identification. Additionally, prior activity recognition investigations revealed that a 10-s window size surpasses others [80].

### 3.3. Data Generation

Data samples are separated into training and test data in this phase, while temporal windows from the signals are utilized to create a model, and test data are used to assess the learned model. Cross-validation is the standard approach for separating data into training and test sets [81]. Numerous strategies, such as k-fold cross-validation [7], could be used to separate the data for training and testing. This stage estimates the learning algorithm’s capacity to generalize to new data. This stage takes advantage of stratified ten-fold cross-validation inside the framework for smartwatch-based user identification. The entire dataset is partitioned into 10 equal folds or subsets for this validation approach. Nine of these folds are utilized for training and one for testing in each cycle. This procedure is performed 10 times, utilizing all data for both training and testing. Stratified data imply that each fold has about the same amount of data from each participant.

### 3.4. The Proposed 1D-ResNet-SE Model

#### 3.4.1. Architecture of the Proposed Model

**Figure 2:**
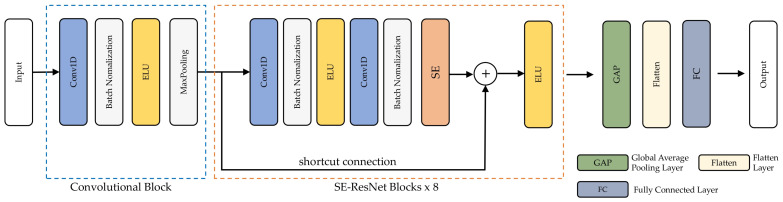
Architecture of the 1D-ResNet-SE.

This work introduces the 1D-ResNet-SE identification model based on complex hand motions acquired by smartwatch sensors to achieve effective biometric user identification. The presented model can automatically extract characteristic features from input sensor data. A convolutional block and SE-ResNet blocks are shown in Figure 2 for capturing spatial features, accompanied by a global average pooling (GAP) layer, and flattened and connected layers directly for further processing.

The input sensors were processed using convolutional blocks and SE-ResNet blocks. An ELU layer, a convolutional layer, a batch normalization layer, and a max-pooling layer were all included in the convolution component. Each of the trainable convolutional kernels in the convolutional layer creates a feature map, which is then used in the convolutional layer. One-dimensional kernels are just like the input spectrum. Because of this, BN was used to stabilize and speed up the learning process. The model’s expression capability was improved with the help of ELU, a nonlinear function. Preserving key characteristics was achieved by using the MP layer to minimize map size. The following section goes into further detail about the SE-ResNet module. Flattened layers were utilized to turn the averages of each feature map into a 1D vector using the GAP. Using a Softmax function, the result of the fully linked layer was transformed into probabilistic reasoning. The cross entropy loss function, which is often used in classification applications, was applied to compute the network’s losses.

#### 3.4.2. SE-ResNet Block

**Figure 3:**
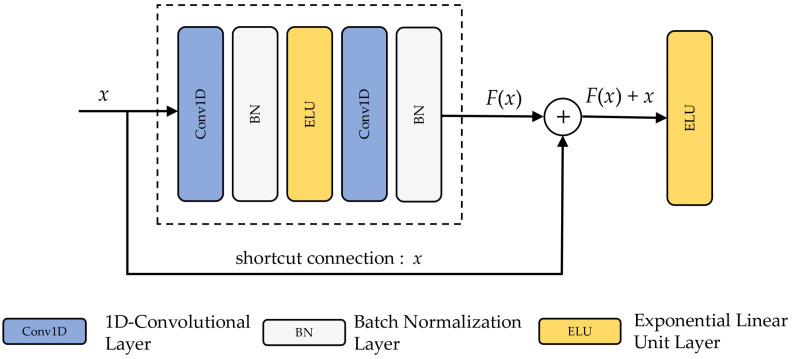
Structure of the residual block.

As the network layers increased, a degradation incident occurred: Accuracy rapidly reached saturation and ultimately declined [82]. Adding a bypass link to ResNet’s residual block could successfully solve the degradation issue [24]. Figure 3 depicts the architecture of a residual block. Convolutional layers, BN, ELU, and a bypass connection are all part of this algorithm. There are no differences between the residual block and the convolutional block except for the bypass link. A residual function *F(x): = H(x) − x* is defined as the sum of the foundation mappings H(x) and F(x) placed on top of each other. Since the initial mapping was transformed into F(x)+x, the initial mapping is no longer relevant. The residual learning is simpler to implement and avoids the degradation issue than simply fitting H(x) using stacked layers.

#### 3.4.3. Squeeze-and-Excitation Module

**Figure 4:**
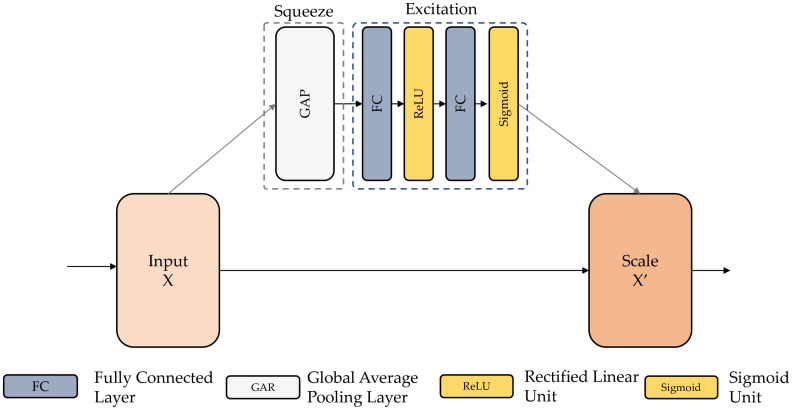
Structure and functionality of the squeeze-and-excitation block.

By combining spatial and channel-specific data, convolutional neural networks extracted the features [83]. The SE module aims to improve the representative capacity of the model’s channel association. After the convolution procedure, many feature maps are obtained. Nonetheless, a few feature maps could well be overloaded with duplication of data. The SE block performs feature recalibration to improve the valuable traits while inhibiting the less valuable ones. Each feature map is squeezed as a first step, and a weight vector is generated. The feature weights are then redistributed using fully connected layers and a sigmoid activation function in the excitation procedure. A gradient descent technique is used to direct the redistribution. Weights are then used to adjust the weights of the features. To recalibrate the feature maps obtained from the stacked layers, the SE block was put behind BN in each residual block in this investigation. Figure 4 depicts the SE-ResNet component’s overall structure and functionality.

#### 3.4.4. Activation Function

The activation function provides a nonlinear element in the model as an essential component. Nonlinear distributed data are challenging to adjust in a network lacking activation functions. Because of this, a network’s capacity to conform to its environment is greatly improved by the activation function. The activation functions that are utilized in this study are as follows.

- Sigmoid function:
  (1) $$\sigma \left(x\right)=\frac{1}{1+e^{-x}}$$
- Rectified Linear Unit (ReLU):
  (2) $$ReLU\left(x\right)=max\left(x,0\right)=\left\{\begin{array}{cc} x & \mathrm{if}\;x\geq 0\\ 0 & \mathrm{if}\;x<0 \end{array}\right.$$
- Exponential Linear Unit (ELU)
  (3) $$ELU\left(x\right)=\left\{\begin{array}{cc} x & \mathrm{if}\;x\geq 0\\ \alpha (e^{x}-1) & \mathrm{if}\;x<0,\mathrm{and}\;\alpha \;\mathrm{defaults}\;\mathrm{to}\;1.0 \end{array}\right.$$

### 3.5. Evaluation Metrics

User identification could be seen as a categorization with many classes. Accuracy, F1-score, and Equal Error Rate are commonly used performance indicators for evaluating and comparing identification systems. These performance indicators are determined using a confusion matrix to determine the model’s ability to detect objects.

**Figure 5:**
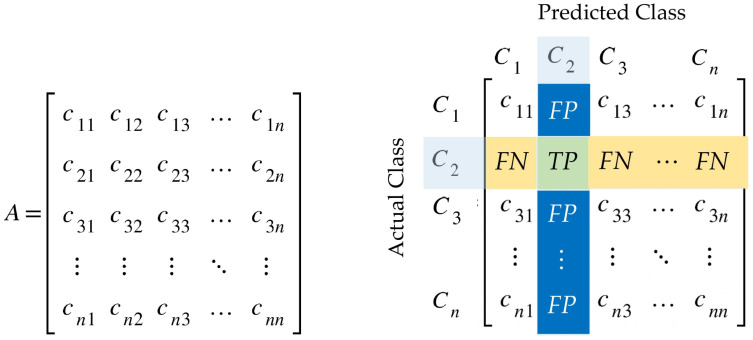
Confusion matrix for a multiclass classification problem.

Let consider a multiclass classification problem with set *A* containing the *n* different class labels $C_{i}\left(i=1,2,3,\ldots ,n\right)$ denoted by $\left\{C_{1},C_{2},C_{3},\ldots ,C_{n}\right\}$. The confusion matrix for that problem is an n×n matrix presented in Figure 5. Each row of the matrix represents the instances of an actual class, while each column represents the instances of a predicted class. An element $C_{ij}$ of the confusion matrix at row *i* and column *j* provides the number of instances for which the actual class is *i* and the predicted class is *j*.

True positive (TP), false positive (FP), true negative (TN), and false negative (FN) are all aspects that could be derived from the confusion matrix and utilized to produce performance metrics. Consider the following mathematical formulae for calculating the label classes $C_{i}$, $TP(C_{i})$, $FP(C_{i})$, $FN(C_{i})$, and $TN(C_{i})$.

(4) $$TP\left(C_{i}\right)=C_{ii}$$

(5) $$FP\left(C_{i}\right)=\sum_{l=1}^{n}C_{li}-TP\left(C_{i}\right)$$

(6) $$FN(C_{i})=\sum_{l=1}^{n}C_{il}-TP\left(C_{i}\right)$$

(7) $$TN\left(C_{i}\right)=\sum_{l=1}^{n}\sum_{k=1}^{n}C_{lk}-TP\left(C_{i}\right)-FP\left(C_{i}\right)-FN\left(C_{i}\right)$$

From Equations (4)–(7), we defined accuracy, precision, recall, and f1-score, for a multiclass confusion matrix in Table 3.

When a biometric-based user identification approach recognizes an invalid individual or fails to realize an actual individual, an error of accuracy happens. False Acceptance Rate (FAR) and False Rejection Rate (FRR) are the most usually exploited measures to determine issues. Equal Error Rate (EER) represents the rate at which FAR and FRR become equal, and hence, a lower EER rate indicates more accuracy.

The typical technique for assessing FAR and FRR for multiclass classifiers is converting the multiclass classification issue to multiple binary classifications. Each class has its own FAR and FRR error values. The EER can be calculated as, $EER=\frac{FAR+FRR}{2}$, where |FAR + FRR| is the smallest value.

## 4. Experimental Results

This section provides the results of all of the experiments we conducted to find the successful deep learning models for sensor-based user identification. The UT-Smoke and WISDM-HARB datasets were used as the two benchmark datasets for person identification utilizing smartwatch sensing data in the research. The accuracy, F1-score, and confusion matrix of the deep learning models were evaluated using these measures.

### 4.1. Software Configuration

Google Colab Pro+ [84] was utilized in this investigation. A graphics processor device called the Tesla V100-SXM2-16GB was used to accelerate the training of the deep learning models. There are several basic deep learning techniques in the Python library, including the 1D-ResNet-SE and Tensorflow backend (version 3.9.1) [85] and CUDA (8.0.6). The following Python libraries were the subject of these explorations:

- When reading, manipulating, and interpreting sensor data, Numpy and Pandas were utilized for data management.
- For plotting and displaying the outcomes of data discovery and model assessment, Matplotlib and Seaborn were utilized.
- Scikit-learn (Sklearn) was used in experiments as a library for sampling and data generation.
- Deep learning models were implemented and trained using TensorFlow, Keras, and TensorBoard.

### 4.2. Experimental Findings

The UT-Smoke and WISDM-HARB datasets were used to validate the developed approach against baseline deep learning methods. These deep learning approaches trained on smartwatch sensing data from the benchmark datasets are described in the following subsections, which give experimental findings. Summary hyperparameters of all models conducted in this study are described in Appendix A.

#### 4.2.1. UT-Smoke

The UT-Smoke dataset was used to collect smartwatch sensor data from 11 participants. Smoking, Eating, Drinking, and Inactive are the four categories of physical activities listed in Table 4. Using classification performance indicators, the evaluated results of the deep learning models were measured (Accuracy and F1 measurements).

Several combinations of smartwatch sensor data and other deep learning techniques, such as CNN and the proposed 1D-ResNet-SE method, can be investigated on the UT-Smoke dataset. To see the categorization results for the DL models mentioned in Table 4, F1 of our recommended 1D-ResNet-SE model derives the score of 97.24% for smoking, 98.13% for eating, and 96.44% for drinking when employing accelerometer, gyroscope, and magnetometer data accordingly. The proposed 1D-ResNet-SE has a greater accuracy and F1 score than existing smartwatch sensor variations. Therefore, we could infer that the approach we propose can recognize smartwatch users quite effectively by employing complex hand movements.

#### 4.2.2. WISDM-HARB

As a second dataset, we used the WISDM-HARB dataset. Smartwatch sensor readings from 44 persons performing 18 physical activities are included in this dataset. This dataset’s classification effectiveness is summarized in Table 5, Table 6 and Table 7.

With the WISDM-HARB dataset, we conducted extensive analysis utilizing two baseline DL models and proposed the 1D-ResNet-SE model. Three separate sensor configurations made use of data from the smartwatch. For ”Clapping” and ”Teeth”, the proposed approach obtained the highest F1 (>95 percent) utilizing both accelerometer and gyroscope data, as shown in Table 5, Table 6 and Table 7.

## 5. Research Discussion

This study aimed to present a deep learning-based framework for identifying users through complicated hand movements using a smartwatch. The proposed approach was evaluated against two distinct benchmark datasets comprising sensor data of various physical human activities acquired by smartwatch motion sensors (accelerometer, gyroscope, and magnetometer). The 1D-ResNet-SE model outperformed previous standard deep learning techniques for smartwatch-based user identification according to experimental outcomes. The 1D-ResNet-SE model uses shortcut connections to resolve the network’s vanishing gradient issue. The proposed model includes SE-ResNet blocks consisting of Conv1D layers, BN layers, ELU layers, squeeze-and-excitation (SE) modules, and a shortcut connection. By combining spatial and channel-specific data, the SE-ResNet block improves identification performance and hierarchically extracts features.

### 5.1. Impact of Squeeze-and-Excitation Modules

It was hypothesized that the squeeze-and-excitation (SE) module might enhance a deep learning model’s channel representational capability. This effort necessitates numerous feature maps and subsequent convolutional procedures. Repetitive information could exist in a few feature maps. The SE module performs feature recalibration to improve the significant attributes while inhibiting the less effective ones. Additional experiments were conducted to compare the introduced 1D-ResNet-SE model versus a modified model that took out the SE component to explore how the SE module affected the results.

To analyze the improvement, a statistical analysis was performed to find out whether there are significant performance differences of accuracy between the baseline 1D-ResNet model and the proposed 1D-ResNet-SE. As suggestion in [86], we perform the Wilcoxon test [87], which is non-parametric statistical test for pairwise comparing the significant difference. In the statistical test, we assume that the null hypothesis $H_{0}$ is as follows: ”There are no significant difference between the model performances”. When performing the non-parametric Wilcoxon test, the null hypothesis that all model performances were equal could be rejected with a significance level of α = 0.05. Hence, the result is statistically significant when *p*-value < 0.05.

Table 8 and Table 9 report the statistical analysis performed via the Wilcoxon test on the UT-Smoke and WISDM-HARB datasets, respectively. Based on the UT-Smoke dataset, the statistical test reveals that the SE module significantly improves accuracy of smartwatch-based user identification using the sensor data of smoking and drinking activities. For statistical analysis based on the WISDM-HARB, the analyzed results reveal similarly that the SE module can improve the user identification using the sensor data of typing, writing, clapping, eating sandwiches, and drinking activities with statistical significance.

### 5.2. Impact of Sensor Combinations

Each sensor’s usefulness to the smartwatch-based user identification is examined in this task. Using accelerometer and gyroscope data as independent inputs, we evaluated the efficiency of the suggested 1D-ResNet-SE model. Utilizing raw accelerometer data with the proposed model resulted in a superior F1 score compared to gyroscope data for all hand-based activities. To analyze the impact of sensor combinations, we utilized the Friedman aligned ranking test [88], which is a non-parametric statistical test for comparing the significant difference. In addition, we applied the Finner post-hoc test [89] with a significance level of α = 0.05 to examine whether the differences in the performance of the model accuracies were statistically significant.

Table 10 and Table 11 present the statistical analyses, performed with non-parametric comparisons that relate to the accuracy metrics of the 1D-ResNet-SE using different sensor data for user identification. The statistical results indicate that the accuracy performance of the 1D-ResNet-SE can be improved significantly by using both accelerometer and gyroscope for smoking, drinking, and eating activities.

### 5.3. Comparison with Previous Works

The recommended 1D-ResNet-SE model is compared to previously trained models on the same dataset (WISDM-HARB). Previous research [17] has revealed that using a machine learning technique called the Random Forest (RF) technique makes it possible to reach high-performance user identification using smartwatch sensors. Prior work presented the stratified 10-fold cross-validation approach, which we employed in our study. Table 12 outlines the comparative findings. The comparison results indicate that the proposed 1D-ResNet-SE model achieved better accuracy than the previous model for most of the activities.

## 6. Conclusions and Future Studies

Using complicated hand gestures and a smartwatch, this study proposes a heterogeneous framework for user identification. Two independent benchmark datasets comprising sensor data from smartwatch motion sensors acquired during diverse individual biological movements were used to evaluate the system (accelerometer, gyroscope, and magnetometer). Three deep learning models were used to classify each dataset’s sensor data, including the standard CNN and LSTM and our proposed 1D-ResNet-SE model.

Metrics such as accuracy and the F-measure were used to determine the experimental outcomes. Classifiers were compared to see how well they performed. Across both datasets, the proposed 1D-ResNet-SE classifier outperformed every other classifier by a wide margin. For user identification, the UT-Smoke dataset delivered high performances from complex hand movements such as eating, smoking, and drinking. We used all three smartwatch sensors (accelerometer, gyroscope, and magnetometer) to classify eating behavior in the UT-Smoke dataset to get a great outcome. Each DL classifier employed in this study performed well with accelerometer data when evaluating its identification capability as a smartwatch sensor. As an alternative, the gyroscope and magnetometer could be utilized to identify individuals. Similar to the WISDM-HARB dataset, the three DL classifiers were examined and assessed using smartwatch sensor data from 18 physical activities. The 1D-ResNet-SE classifier surpassed the other baseline DL classifiers in the investigation. User identification also gave valuable insights into the nature of users’ actions. Using a smartwatch to identify a user was an effective solution for this kind of action.

Even though the existing smartwatch-based user identification sensor method achieves good results, future studies might benefit from researching different replacements for the proposed solution. Another option is to include a wide range of activities, such as more complicated and transitional tasks, within a systematic framework to increase user identification. In the future, a complete smartphone and smartwatch dataset could be evaluated that includes numerous body locations for smartphone placements, since the smartwatch sensor data are only investigated in one position in the current study. Position-based user identification can be used to enhance identification outcomes in this manner.

## Figures and Tables

**Table 1:** A summary of existing literature on user identification based on the sensor data.

Work (Year)	Classifier	Sensors	Device	Performance (% Accuracy)	Contribution	No. of Users
Parziale et al.(2021) [44]	Random Forest	1 Acc.	Smartwatch	89.77	User identification based on writing activity performed in air	98
Mekruksavanich et al. (2021) [20]	CNN-LSTM	2 Acc. 1 Gyro.	Smartphone	94.57	User identification based on smartphone sensor from dynamic activities (walking, walking upstairs, and walking downstairs)	30
Benegui et al. (2020) [21]	CNN	1 Acc. 1 Gyro.	Smartphone	90.75	User identification based on motion sensor data of tapping on screen motion from smartphone	50
Angrisano et al. (2020) [19]	Random Forest	1 Acc. 1 Gyro.	Smartphone	93.8	User identification based on walking activities using ensemble machine learning	32
Weiss et al. (2019) [17]	Random Forest	1 Acc. 1 Gyro.	Smartphone	92.7	User identification based on simple activities and complex activities using machine learning approaches	51
		1 Acc. 1 Gyro.	Smartwatch	71.7		
Musale et al. (2019) [45]	Random Forest	1 Acc. 1 Gyro.	Smartwatch	91.8	User identification based on statistical features and human-action-related features from sensor data	51
Ahmad et al. (2018) [18]	Decision Tree	1 Acc. 1 Gyro. 1 Mag.	Smartwatch	98.68	User identification based on ambulatory activities using machine learning	6
Nevero et al. (2016) [22]	CNN	1 Acc. 1 Gyro. 1 Mag.	Smartphone	69.41	User identification based on walking activities using dense convolutional clockwork RNNs	587

**Table 2:** Characteristics of the selected activity-based datasets.

Dataset	Category	Activity	Description	Raw Sensor Data	Percentage
UT-Smoke	Simple	Sitting	Sitting	649,000	14.28%
		Standing	Standing	649,000	14.29%
	Complex	Smoking	Smoking	1,298,000	28.57%
		Eating	Eating	649,000	14.29%
		Drinking	Drinking	1,298,000	28.57%
WISDM-HARB	Simple	Walking	Walking	192,531	5.60%
		Jogging	Jogging	187,833	5.46%
		Stairs	Walking upstairs and downstairs	180,416	5.24%
		Sitting	Sitting	195,050	5.67%
		Standing	Standing	194,103	5.64%
		Kicking	Kicking a soccer ball	191,535	5.57%
	Complex	Dribbling	Dribbling a basketball	194,845	5.66%
		Catch	Playing catch a tennis ball	187,684	5.46%
		Typing	Typing	187,175	5.44%
		Writing	Writing	197,403	5.74%
		Clapping	Clapping	190,776	5.55%
		Teeth	Brushing teeth	190,759	5.54%
		Folding	Folding clothes	193,373	5.62%
		Pasta	Eating pasta	189,609	5.51%
		Soup	Eating soup	187,057	5.44%
		Sandwich	Eating a sandwich	190,191	5.53%
		Chips	Eating chips	192,085	5.58%
		Drinking	Drinking from a cup	197,917	5.75%

**Table 3:** Performance metrics for a multiclass confusion matrix.

Metrics	Formulas
Accuracy	$Accuracy=\frac{\sum_{i=1}^{n}TP\left(C_{i}\right)}{\sum_{i=1}^{n}\sum_{j=1}^{n}c_{ij}}$
Recall of class $C_{i}$	$$Recall\left(C_{i}\right)=\frac{TP(C_{i})}{TP\left(C_{i}\right)+FN\left(C_{i}\right)}$$
Precision of class $C_{i}$	$$Precision\left(C_{i}\right)=\frac{TP(C_{i})}{TP\left(C_{i}\right)+FP\left(C_{i}\right)}$$
F1-score of class $C_{i}$	$$F1-score\left(C_{i}\right)=2\times \frac{Precision\left(C_{i}\right)\times Recall\left(C_{i}\right)}{Precision\left(C_{i}\right)+Recall\left(C_{i}\right)}$$
Recall	$Recall=\frac{1}{n}\sum_{i=1}^{n}Recall\left(C_{i}\right)$
Precision	$Precision=\frac{1}{n}\sum_{i=1}^{n}Precision\left(C_{i}\right)$
F1-score	$F1-score=2\times \frac{Precision\times Recall}{Precision+Recall}$
False Acceptance Rate of class $C_{i}$	$$FAR\left(C_{i}\right)=\frac{FN(C_{i})}{FN\left(C_{i}\right)+TP\left(C_{i}\right)}$$
False Rejection Rate of class $C_{i}$	$$FRR\left(C_{i}\right)=\frac{FP(C_{i})}{FP\left(C_{i}\right)+TN\left(C_{i}\right)}$$

**Table 4:** Identification effectiveness on classifier evaluation of deep learning models using various combinations of sensor data from UT-Smoke dataset.

Sensor	Activity	Recognition Achievement of DL Models Using Sensors Data from UT-Smoke Dataset								
		CNN			LSTM			1D-ResNet-SE		
		Accuracy ± SD	F1 ± SD	EER	Accuracy ± SD	F1 ± SD	EER	Accuracy ± SD	F1 ± SD	EER
Acc.	Smoking	69.42 (±0.530)	69.35 (±0.545)	32.41(±1.17)	65.47 (±3.885)	65.00 (±4.409)	31.26 (±1.12)	89.06 (±0.560)	89.04 (±0.577)	13.49 (±0.87)
	Eating	80.85 (±0.523)	80.64 (±0.602)	21.22 (±0.70)	79.92 (±2.653)	79.81 (±3.136)	18.35 (±1.26)	88.33 (±5.448)	88.20 (±5.608)	14.60 (±1.29)
	Drinking	66.41 (±1.574)	65.86 (±1.746)	33.20 (±1.35)	66.20 (±2.704)	65.75 (±3.436)	43.19 (±1.25)	84.81 (±1.052)	84.77 (±1.090)	15.14 (±1.08)
	Inactive	14.61 (±0.560)	5.54 (±1.068)	91.19 (±0.32)	14.82 (±0.109)	6.77 (±0.564)	90.66 (±0.41)	14.54 (±0.273)	8.01 (±1.120)	90.44 (±0.56)
	Avg. (all)	57.82	55.35	44.51	56.60	54.33	45.87	69.19	67.53	33.42
	Avg. (active)	72.23	71.95	28.94	70.53	70.19	30.93	87.40	87.34	14.41
Gyro.	Smoking	51.28 (±0.380)	51.07 (±0.418)	50.53 (±0.69)	45.81 (±0.649)	45.19 (±1.065)	56.99 (±1.86)	68.64 (±5.345)	68.63 (±4.833)	33.74 (±7.32)
	Eating	67.03 (±0.394)	66.36 (±0.431)	35.29 (±1.27)	61.62 (±1.548)	59.92 (±1.717)	44.20 (±2.25)	80.42 (±4.736)	80.77 (±4.417)	21.07 (±7.20)
	Drinking	43.00 (±0.657)	42.67 (±0.515)	57.71 (±0.54)	37.09 (±3.286)	34.79 (±3.426)	65.06 (±2.30)	46.40 (±5.949)	45.72 (±6.773)	54.97 (±3.14)
	Inactive	11.49 (±0.650)	7.03 (±0.904)	92.97 (±0.42)	15.08 (±0.168)	4.40 (±0.250)	91.14 (±0.05)	13.11 (±0.705)	7.21 (±0.479)	90.80 (±0.35)
	Avg. (all)	43.20	41.78	59.13	39.90	36.08	64.35	52.14	50.58	50.15
	Avg. (active)	53.77	53.37	47.84	48.17	46.63	55.42	65.15	65.04	36.59
Mag.	Smoking	54.77 (±3.998)	53.79 (±4.619)	48.86 (±2.92)	57.85 (±2.779)	56.77 (±3.134)	40.62 (±1.43)	78.06 (±10.695)	78.18 (±10.496)	21.49 (±8.35)
	Eating	76.10 (±3.206)	76.10 (±3.163)	25.01 (±1.02)	78.77 (±2.733)	78.34 (±2.959)	22.21 (±3.35)	89.81 (±3.266)	89.85 (±3.194)	15.08 (±5.63)
	Drinking	44.09 (±4.993)	42.58 (±6.440)	53.45 (±3.64)	63.66 (±1.984)	63.69 (±2.074)	40.68 (±1.62)	76.82 (±7.711)	76.92 (±7.568)	24.07 (±4.91)
	Inactive	15.41 (±0.006)	4.11 (±0.003)	90.92 (±0.02)	14.59 (±0.230)	8.64 (±1.611)	90.43 (±0.51)	14.34 (±0.343)	8.69 (±1.485)	89.95 (±0.67)
	Avg. (all)	47.59	44.15	54.56	53.72	51.86	48.49	64.76	63.41	37.65
	Avg. (active)	58.32	57.49	42.44	66.76	66.27	34.50	81.56	81.65	20.21
Acc.+ Gyro.	Smoking	71.11 (±0.928)	70.92 (±1.067)	29.13 (±0.78)	69.24 (±2.088)	69.29 (±2.119)	9.58 (±1.61)	91.20 (±0.610)	91.19 (±0.612)	9.74 (±1.94)
	Eating	83.27 (±0.420)	83.10 (±0.425)	17.74 (±1.23)	84.12 (±2.734)	84.42 (±2.471)	15.51 (±0.38)	91.65 (±1.781)	91.69 (±1.756)	8.42 (±1.66)
	Drinking	67.92 (±0.905)	67.58 (±1.027)	32.90 (±1.46)	65.87 (±3.575)	66.06 (±3.352)	31.38 (±2.72)	84.78 (±2.265)	84.73 (±2.367)	13.63 (±0.41)
	Inactive	14.21 (±0.829)	6.77 (±1.379)	91.48 (±0.34)	14.61 (±0.352)	8.01 (±0.966)	90.96 (±0.11)	14.14 (±0.608)	8.47 (±1.316)	90.24 (±0.65)
	Avg. (all)	59.13	57.09	42.81	58.46	56.95	36.86	70.44	69.02	30.51
	Avg. (active)	74.10	73.87	26.59	73.08	73.26	18.82	89.21	89.20	10.60
Acc.+Mag.	Smoking	87.14 (±1.329)	87.14 (±1.329)	11.82 (±0.98)	82.27 (±2.381)	82.24 (±2.449)	18.64 (±2.91)	96.63 (±0.489)	96.63 (±0.490)	3.07 (±0.51)
	Eating	96.08 (±0.462)	96.07 (±0.449)	4.81 (±0.58)	93.34 (±3.324)	93.40 (±3.181)	5.66 (±2.15)	97.98 (±0.088)	97.96 (±0.082)	2.88 (±0.79)
	Drinking	86.80 (±0.974)	86.90 (±0.906)	12.66 (±1.00)	78.65 (±6.253)	78.69 (±6.262)	20.23 (±4.56)	96.47 (±0.237)	96.46 (±0.245)	3.41 (±0.36)
	Inactive	15.26 (±0.181)	4.54 (±0.710)	90.94 (±0.04)	14.81 (±0.197)	7.05 (±0.460)	89.98 (±0.42)	14.54 (±0.481)	8.73 (±0.651)	8.38 (± 1.82)
	Avg. (all)	71.32	90.04	30.06	67.27	84.78	33.63	76.41	74.95	4.44
	Avg. (active)	90.01	54.93	9.76	84.75	64.97	14.84	97.03	97.02	3.12
Gyro.+Mag.	Smoking	60.64 (±1.497)	60.54 (±1.645)	40.23 (±3.16)	61.35 (±2.468)	60.59 (±2.604)	38.22 (±4.16)	86.76 (±3.827)	86.79 (±3.738)	17.74 (±4.98)
	Eating	81.14 (±1.512)	81.17 (±1.449)	20.61 (±1.47)	81.67 (±1.875)	81.50 (±1.917)	20.29 (±1.46)	87.24 (±4.701)	87.52 (±4.434)	7.71 (±1.80)
	Drinking	49.13 (±16.952)	46.86 (±21.449)	47.88 (±3.49)	63.93 (±2.815)	63.79 (±2.591)	40.80 (±2.80)	78.58 (±2.384)	78.60 (±2.304)	20.04 (±4.20)
	Inactive	15.10 (±0.577)	4.61 (±0.967)	90.91 (±0.01)	15.01 (±0.206)	6.34 (±1.098)	90.63 (±0.23)	14.43 (±0.162)	8.56 (±1.152)	89.93 (±0.45)
	Avg. (all)	51.50	48.30	49.91	68.98	53.06	47.49	66.75	65.37	33.86
	Avg. (active)	63.64	62.86	36.24	44.39	68.63	33.10	84.19	84.30	15.16
Acc.+Gyro.+Mag.	Smoking	88.68 (±0.492)	88.68 (±0.477)	11.60 (±1.10)	80.40 (±2.335)	80.39 (±2.450)	21.50 (±6.94)	97.24 (±0.280)	97.24 (±0.280)	2.85 (±0.46)
	Eating	95.84 (±1.247)	95.83 (±1.246)	5.02 (±0.57)	95.18 (±0.658)	95.18 (±0.613)	5.93 (±2.22)	98.15 (±0.178)	98.13 (±0.179)	2.32 (±0.27)
	Drinking	88.16 (±0.916)	88.27 (±0.888)	14.15 (±1.65)	80.18 (±4.504)	80.36 (±4.613)	19.53 (±3.39)	96.54 (±0.511)	96.54 (±0.496)	3.39 (±0.22)
	Inactive	15.34 (±0.136)	4.13 (±0.038)	90.90 (±0.01)	14.89 (±0.325)	8.38 (±1.965)	90.57 (±0.56)	14.62 (±0.330)	9.22 (±1.107)	89.92 (±0.68)
	Avg. (all)	72.01	69.23	30.42	67.66	66.08	34.38	76.64	75.28	24.62
	Avg. (active)	90.89	90.93	10.26	85.25	85.31	15.65	97.31	97.30	2.85

**Table 5:** Recognition effectiveness on classifier evaluation of deep learning models using WIDSM-HARB dataset (Acc. and Gyro. sensors).

	Activity	Identification Performance on Classifier Evaluation of DL Models Using WIDSM-HARB Dataset (Acc. and Gyro.).								
		CNN			LSTM			1D-ResNet-SE		
		Accuracy ± SD	F1 ± SD	EER	Accuracy ± SD	F1 ± SD	EER	Accuracy ± SD	F1 ± SD	EER
**Simple Motion**	Walking	68.14 (±1.230)	67.41 (±1.295)	31.35 (±3.92)	78.00 (±3.059)	77.30 (±3.504)	20.84 (±3.30)	93.26 (±3.302)	93.35 (±3.131)	10.82 (±7.16)
	Jogging	74.66 (±2.131)	73.84 (±2.309)	27.69 (±2.16)	86.84 (±2.473)	86.62 (±2.518)	14.74 (±2.32)	96.25 (±2.100)	96.20 (±2.088)	2.43 (±0.49)
	Stairs	41.84 (±3.244)	42.13 (±2.911)	56.01 (±2.29)	58.04 (±3.625)	56.90 (±3.718)	44.54 (±2.57)	82.83 (±7.179)	82.53 (±7.345)	13.01 (±8.27)
	Sitting	68.47 (±1.366)	67.62 (±1.484)	31.42 (±1.68)	67.03 (±2.887)	64.80 (±2.883)	33.28 (±3.25)	71.66 (±4.868)	69.90 (±5.767)	25.47 (±5.56)
	Standing	59.47 (±2.546)	58.81 (±2.539)	41.81 (±1.63)	61.53 (±1.138)	58.65 (±1.455)	38.48 (±2.43)	64.12 (±8.317)	61.68 (±9.090)	36.11 (±4.95)
	Kicking	37.53 (±2.361)	36.92 (±1.994)	64.52 (±3.56)	46.56 (±2.854)	43.75 (±3.225)	54.15 (±1.65)	84.28 (±5.572)	84.36 (±5.362)	17.35 (±8.92)
**Hand Complex Movement**	Dribbling	58.55 (±6.655)	57.98 (±6.575)	40.81 (±2.00)	75.24 (±1.676)	74.43 (±1.958)	26.65 (±2.45)	93.43 (±4.231)	93.35 (±4.284)	9.43 (±11.91)
	Catch	52.55 (±3.207)	51.03 (±3.199)	53.87 (±3.78)	64.22 (±4.014)	62.49 (±4.472)	33.74 (±4.20)	94.37 (±4.718)	94.31 (±4.806)	4.89 (±1.39)
	Typing	76.98 (±1.429)	76.26 (±1.701)	22.08 (±1.56)	70.74 (±4.555)	67.79 (±5.454)	28.46 (±2.52)	84.69 (±2.886)	83.74 (±3.188)	18.34 (±3.93)
	Writing	72.06 (±2.128)	71.10 (±2.416)	32.28 (±2.80)	70.23 (±1.821)	68.06 (±2.037)	31.26 (±3.88)	81.67 (±8.008)	81.10 (±8.612)	38.34 (±20.69)
	Clapping	78.59 (±3.466)	77.96 (±4.025)	17.48 (±3.24)	88.89 (±2.398)	88.55 (±2.590)	12.69 (±1.32)	95.99 (±2.523)	95.76 (±2.800)	3.14 (±2.36)
	Teeth	68.09 (±2.737)	67.14 (±3.101)	31.68 (±2.35)	68.64 (±4.315)	67.29 (±4.261)	29.63 (±2.89)	95.31 (±1.966)	95.16 (±2.079)	5.31 (±1.57)
	Folding	37.80 (±1.622)	36.31 (±1.761)	59.93 (±2.56)	48.84 (±2.396)	46.15 (±2.343)	51.28 (±1.90)	76.12 (±11.614)	75.19 (±12.463)	22.84 (±8.31)
	Pasta	56.11 (±2.703)	55.01 (±2.727)	43.67 (±1.52)	62.89 (±2.473	61.09 (±2.401)	37.88 (±2.83)	82.81 (±5.391)	82.50 (±5.598)	15.31 (±4.55)
	Soup	64.68 (±2.369)	64.21 (±2.512)	34.25 (±2.91)	71.31 (±2.613)	69.99 (±3.035)	30.06 (±0.76)	88.02 (±6.895)	87.92 (±7.091)	10.69 (±1.49)
	Sandwich	50.83 (±2.568)	49.43 (±2.252)	47.98 (±4.74)	56.83 (±1.992)	53.25 (±2.512)	45.50 (±1.13)	78.08 (±1.063)	77.83 (±0.953)	21.99 (±2.49)
	Chips	50.52 (±2.702)	49.53 (±2.206)	52.60 (±2.54)	58.42 (±3.183)	55.94 (±3.239)	38.07 (±2.41)	81.20 (±6.487)	80.88 (±6.662)	16.35 (±2.72)
	Drinking	60.11 (±2.413)	59.51 (±2.591)	40.90 (±4.19)	61.94 (±2.148)	59.57 (±2.682)	37.74 (±1.33)	81.80 (±1.485)	81.38 (±1.675)	17.02 (±1.90)
	Average	59.83	59.01	40.57	66.45	64.59	34.94	84.77	84.29	16.05

**Table 6:** Recognition effectiveness on classifier evaluation of deep learning models using WIDSM-HARB dataset (Acc. sensor).

	Activity	Recognition Effectiveness on Classifier Evaluation of DL Models Using WIDSM-HARB Dataset (Acc.).								
		CNN			LSTM			1D-ResNet-SE		
		Accuracy ± SD	F1 ± SD	EER	Accuracy ± SD	F1 ± SD	EER	Accuracy ± SD	F1 ± SD	EER
**Simple Motion**	Walking	52.14 (±1.384)	52.16 (±1.541)	44.87 (±3.05)	66.05 (±2.426)	64.99 (±3.000)	38.45 (±4.01)	91.91 (±1.623)	91.65 (±1.755)	12.01 (±4.26)
	Jogging	54.80 (±3.332)	54.32 (±3.556)	46.36 (±3.85)	77.74 (±3.976)	77.48 (±4.231)	25.76 (±2.69)	93.42 (±1.980)	93.28 (±2.238)	7.10 (±2.47)
	Stairs	32.28 (±2.522)	31.01 (±2.780)	66.85 (±1.16)	45.61 (±3.929)	42.67 (±4.630)	52.93 (±2.56)	78.01 (±8.410)	77.24 (±8.826)	20.46 (±7.40)
	Sitting	64.98 (±2.314)	64.45 (±2.472)	35.50 (±1.93)	60.35 (±3.066)	57.48 (±3.784)	39.59 (±2.06)	71.55 (±5.009)	70.41 (±5.788)	28.85 (±3.33)
	Standing	61.24 (±1.972)	59.61 (±2.221)	38.37 (±2.54)	56.53 (±2.148)	52.08 (±2.355)	43.65 (±1.15)	62.47 (±3.619)	60.96 (±3.654)	36.99 (±5.82)
	Kicking	28.93 (±2.614)	28.05 (±2.555)	68.49 (±1.20)	41.58 (±2.884)	38.70 (±3.460)	59.30 (±3.39)	72.67 (±5.045)	71.68 (±5.070)	27.05 (±2.53)
**Hand Complex Movement**	Dribbling	44.34 (±1.771)	43.48 (±1.764)	54.72 (±1.35)	63.01 (±6.270)	61.11 (±6.266)	38.50 (±1.26)	90.96 (±3.829)	90.86 (±3.856)	12.20 (±14.56)
	Catch	37.40 (±3.813)	36.62 (±3.898)	65.13 (±2.53)	57.04 (±4.027)	55.43 (±4.389)	42.97 (±2.62)	90.66 (±1.680)	90.72 (±1.544)	19.85 (±14.15)
	Typing	70.12 (±7.315)	68.85 (±7.755)	29.26 (±4.93)	68.77 (±2.418)	65.68 (±2.560)	2.18 (±2.71)	77.22 (±5.938)	75.81 (±6.881)	25.46 (±7.33)
	Writing	64.71 (±1.891)	64.06 (±2.342)	37.97 (±3.06)	63.23 (±4.084)	60.84 (±4.719)	37.34 (±3.96)	54.76 (±16.852)	52.27 (±17.678)	35.14 (±10.76)
	Clapping	71.63 (±5.277)	70.78 (±5.572)	29.46 (±2.11)	79.82 (±3.143)	79.16 (±3.505)	20.45 (±2.23)	93.09 (±2.551)	93.00 (±2.561)	7.03 (±2.25)
	Teeth	59.26 (±2.407)	58.51 (±2.259)	37.73 (±3.23)	63.27 (±2.111)	61.47 (±1.890)	38.69 (±3.05)	90.37 (±3.344)	90.34 (±3.345)	7.91 (±1.38)
	Folding	36.24 (±3.944)	35.30 (±3.842)	63.66 (±2.34)	44.42 (±0.754)	41.62 (±0.719)	54.68 (±4.95)	79.54 (±3.411)	79.24 (±3.461)	22.39 (±2.81)
	Pasta	52.12 (±2.994)	51.58 (±2.995)	48.38 (±2.25)	56.60 (±2.922)	54.34 (±3.274)	43.61 (±3.34)	82.39 (±2.943)	81.93 (±2.987)	27.56 (±11.70)
	Soup	59.27 (±3.055)	58.43 (±3.125)	42.73 (±4.47)	60.63 (±4.157)	58.12 (±4.833)	39.18 (±1.01)	82.68 (±6.134)	81.78 (±7.039)	17.38 (±7.21)
	Sandwich	50.46 (±2.102)	49.33 (±1.517)	50.20 (±2.12)	53.28 (±1.784)	50.06 (±1.866)	23.80 (±1.76)	74.28 (±1.979)	73.70 (±2.101)	23.80 (±1.76)
	Chips	47.76 (±2.208)	46.53 (±2.561)	55.04 (±3.66)	52.29 (±2.488)	50.14 (±2.893)	24.22 (±2.76)	76.91 (±1.069)	76.47 (±1.202)	24.22 (±2.76)
	Drinking	55.08 (±2.754)	54.03 (±2.571)	45.96 (±2.47)	56.44 (±1.570)	53.36 (±1.842)	24.81 (±3.13)	74.23 (±3.473)	73.21 (±3.614)	24.81 (±3.13)
	Average	52.38	51.51	47.82	59.26	53.93	36.12	79.84	79.14	21.12

**Table 7:** Recognition effectiveness on classifier evaluation of deep learning models using WIDSM-HARB dataset. (Gyro. sensor).

	Activity	Recognition Effectiveness on Classifier Evaluation of DL Models Using WIDSM-HARB Dataset (Gyro.).								
		CNN			LSTM			1D-ResNet-SE		
		Accuracy ± SD	F1 ± SD	EER	Accuracy ± SD	F1 ± SD	EER	Accuracy ± SD	F1 ± SD	EER
**Simple Motion**	Walking	54.72 (±1.465)	54.18 (±1.506)	45.97 (±1.50)	49.69 (±4.723)	46.49 (±4.400)	51.33 (±4.89)	84.74 (±13.344)	83.92 (±14.802)	7.89 (±3.86)
	Jogging	58.73 (±2.690)	57.80 (±2.572)	42.06 (±2.87)	61.87 (±4.805)	59.95 (±5.992)	38.91 (±4.88)	92.74 (±11.612)	92.15 (±12.797)	8.80 (±6.26)
	Stairs	23.57 (±1.631)	22.37 (±1.524)	76.48 (±1.59)	24.48 (±3.510)	21.656 (±2.971)	75.56 (±3.52)	83.57 (±8.468)	83.22 (±8.659)	15.12 (±6.85)
	Sitting	18.53 (±1.513)	17.97 (±1.147)	82.53 (±1.63)	09.57 (±0.354)	7.31 (±0.710)	91.07 (±0.29)	19.61 (±1.308)	18.00 (±1.280)	81.55 (±2.27)
	Standing	14.76 (±2.124)	14.22 (±2.384)	86.09 (±1.98)	8.118 (±0.802)	05.87 (±1.002)	92.83 (±1.15)	20.76 (±6.836)	19.57 (±7.979)	73.52 (±8.14)
	Kicking	17.07 (±2.259)	16.01 (±2.424)	83.26 (±1.97)	25.31 (±4.754)	22.65 (±4.271)	75.62 (±4.49)	80.47 (±5.510)	80.06 (±5.909)	18.14 (±3.50)
**Hand Complex Movement**	Dribbling	49.88 (±1.678)	48.88 (±1.400)	50.08 (±1.88)	60.96 (±4.594)	58.01 (±5.165)	39.32 (±4.55)	91.75 (±11.119)	91.51 (±11.570)	3.30 (±1.73)
	Catch	33.93 (±1.015)	33.61 (±0.900)	66.97 (±0.84)	32.37 (±5.311)	29.080 (±5.411)	68.47 (±5.36)	92.457 (±7.628)	92.17 (±8.210)	3.76 (±0.92)
	Typing	26.54 (±1.069)	25.02 (±1.136)	74.55 (±1.22)	33.27 (±3.089)	29.61 (±3.073)	67.45 (±3.26)	82.84 (±11.221)	82.54 (±11.622)	25.63 (±6.83)
	Writing	23.84 (±1.954)	22.86 (±2.152)	78.04 (±1.46)	34.76 (±5.479)	32.16 (±5.968)	66.74 (±5.36)	58.64 (±15.074)	54.18 (±17.788)	25.54 (±20.07)
	Clapping	77.48 (±2.574)	77.14 (±2.449)	22.94 (±2.61)	73.66 (±1.376)	71.98 (±1.735)	26.95 (±1.46)	95.37 (±6.640)	95.20 (±6.964)	3.77 (±2.49)
	Teeth	49.38 (±1.549)	48.38 (±1.764)	50.47 (±1.48)	40.86 (±4.84)	37.24 (±4.594)	59.50 (±4.63)	92.65 (±2.852)	92.57 (±2.887)	11.24 (±5.35)
	Folding	11.67 (±1.063)	11.21 (±0.950)	88.86 (±0.97)	14.41 (±2.488)	12.81 (±2.683)	86.88 (±2.37)	74.17 (±1.693)	73.70 (±2.128)	25.83 (±7.36)
	Pasta	23.67 (±2.899)	22.61 (±3.101)	77.48 (±2.74)	15.32 (±1.698)	11.90 (±1.699)	85.23 (±1.69)	81.90 (±2.340)	81.38 (±2.690)	14.71 (±2.77)
	Soup	34.58 (±2.684)	33.87 (±2.541)	65.77 (±2.71)	26.41 (±4.782)	22.86 (±4.585)	74.54 (±4.90)	85.69 (±7.019)	85.09 (±7.844)	11.14 (±9.50)
	Sandwich	19.41 (±0.922)	18.56 (±0.911)	80.71 (±0.69)	16.10 (±2.210)	12.42 (±2.770)	84.23 (±2.25)	53.65 (±9.941)	51.81 (±10.759)	48.61 (±17.12)
	Chips	18.25 (±2.270)	17.57 (±1.829)	82.18 (±2.31)	14.88 (±4.596)	11.26 (±4.318)	85.71 (±4.51)	73.12 (±10.084)	73.25 (±10.004)	27.18 (±4.45)
	Drinking	25.06 (±1.836)	23.83 (±1.812)	76.07 (±1.79)	17.44 (±8.376)	14.10 (±8.025)	83.43 (±8.37)	55.03 (±15.631)	53.43 (±16.501)	48.35 (±14.77)
	Average	32.28	31.45	68.36	31.08	28.19	66.02	73.29	72.43	25.23

**Table 8:** Wilcoxon test based on the accuracy metrics for model performance comparison on the UT-Smoke dataset.

Hand Complex Movement	Test Models	Accuracy ± SD	Wilcoxon Test	
			*p*-Value	$H_{0}$
Smoking	1D-ResNet	93.61 (±0.45)	0.043	reject
	1D-ResNet-SE	97.24 (±0.28)		
Eating	1D-ResNet	97.19 (±0.21)	0.079	accept
	1D-ResNet-SE	98.15 (±0.18)		
Drinking	1D-ResNet	92.71 (±0.51)	0.022	reject
	1D-ResNet-SE	96.62 (±0.33)		

**Table 9:** Wilcoxon test based on the accuracy metrics for model performance comparison on the WISDM-HARB dataset.

Hand Complex Movement	Test Models	Accuracy ± SD	Wilcoxon Test	
			*p*-Value	$H_{0}$
Dribbling	1D-ResNet	92.95 (±1.07)	0.686	accept
	1D-ResNet-SE	93.43 (±4.23)		
Catch	1D-ResNet	94.36 (±0.58)	0.691	accept
	1D-ResNet-SE	94.37 (±4.12)		
Typing	1D-ResNet	76.67 (±13.19)	0.039	reject
	1D-ResNet-SE	84.69 (±2.89)		
Writing	1D-ResNet	53.30 (±15.65)	0.012	reject
	1D-ResNet-SE	81.67 (±8.01)		
Clapping	1D-ResNet	94.78 (±0.93)	0.043	reject
	1D-ResNet-SE	95.99 (±2.52)		
Teeth	1D-ResNet	95.00 (±2.66)	0.642	accept
	1D-ResNet-SE	95.31 (±1.97)		
Folding	1D-ResNet	75.41 (±2.57)	0.633	accept
	1D-ResNet-SE	76.12 (±11.61)		
Pasta	1D-ResNet	81.01 (±2.44)	0.345	accept
	1D-ResNet-SE	82.81 (±5.39)		
Soup	1D-ResNet	87.35 (±2.08)	0.728	accept
	1D-ResNet-SE	88.02 (±6.90)		
Sandwich	1D-ResNet	77.83 (±2.80)	0.043	reject
	1D-ResNet-SE	78.08 (±1.06)		
Chips	1D-ResNet	80.40 (±5.86)	0.138	accept
	1D-ResNet-SE	81.20 (±6.49)		
Drinking	1D-ResNet	78.86 (±4.68)	0.025	reject
	1D-ResNet-SE	81.80 (±1.49)		

**Table 10:** Friedman aligned ranking test and Finner post-hoc test based on the accuracy metrics of the 1D-ResNet-SE model using different sensor data on the UT-Smoke dataset.

Activity	Models	Friedman Aligned Ranking Test	Finner Post-Hoc Test	
			*p*-Value	$H_{0}$
Smoking	Acc.+Gyro.	1.1526	-	-
	Acc.	2.6264	0.0000021	reject
	Gyro.	4.3519	0.0000203	reject
Eating	Acc.+Gyro.	1.2728	-	-
	Acc.	2.6168	0.0000036	reject
	Gyro.	4.5962	0.0016952	reject
Drinking	Acc.+Gyro.	1.0607	-	-
	Acc.	2.8284	0.0000001	reject
	Gyro.	4.0607	0.0000001	reject

**Table 11:** Friedman aligned ranking test and Finner post-hoc test based on the accuracy metrics of the 1D-ResNet-SE model using different sensor data on the WISDM-HARB dataset.

Activity	Models	Friedman Aligned Ranking Test	Finner Post-Hoc Test	
			*p*-Value	$H_{0}$
Dribbling	Acc.+Gyro.	1.7678	-	-
	Acc.	3.3941	0.346	accept
	Gyro.	3.3234	0.786	accept
Catch	Acc.+Gyro.	1.0606	-	-
	Acc.	3.8890	0.043	reject
	Gyro.	3.5355	0.225	accept
Typing	Acc.+Gyro.	1.9091	-	-
	Acc.	3.6769	0.345	accept
	Gyro.	2.8991	0.686	accept
Writing	Acc.+Gyro.	2.1920	-	-
	Acc.	3.6062	0.501	accept
	Gyro.	2.6870	0.345	accept
Clapping	Acc.+Gyro.	1.3435	-	-
	Acc.	3.8184	0.079	accept
	Gyro.	3.3234	0.893	accept
Teeth	Acc.+Gyro.	1.6971	-	-
	Acc.	2.7577	0.043	reject
	Gyro.	4.0305	0.138	accept
Folding	Acc.+Gyro.	2.9698	-	-
	Acc.	2.4041	0.982	accept
	Gyro.	3.1113	0.502	accept
Pasta	Acc.+Gyro.	1.4142	-	-
	Acc.	3.4648	0.079	accept
	Gyro.	3.6062	0.892	accept
Soup	Acc.+Gyro.	1.8384	-	-
	Acc.	3.5355	0.041	reject
	Gyro.	3.1112	0.501	accept
Sandwich	Acc.+Gyro.	1.0607	-	-
	Acc.	3.5355	0.138	accept
	Gyro.	3.8891	0.042	reject
Chips	Acc.+Gyro.	1.4849	-	-
	Acc.	2.4042	0.041	reject
	Gyro.	4.5962	0.015	reject
Drinking	Acc.+Gyro.	1.1313	-	-
	Acc.	3.1113	0.043	reject
	Gyro.	4.2426	0.021	reject

**Table 12:** The comparison results of the proposed 1D-ResNet-SE model and the previous work model.

	Activity	Identification Performance (%Accuracy)					
		Acc.		Gyro.		Acc.+Gyro.	
		Random Forest [17]	1D-ResNet-SE	Random Forest [17]	1D-ResNet-SE	Random Forest [17]	1D-ResNet-SE
**Simple Motion**	Walking	75.10	91.91	67.00	84.74	78.90	93.26
	Jogging	75.00	93.42	74.30	92.74	82.10	96.25
	Stairs	52.40	78.01	39.20	83.57	58.70	82.83
	Sitting	70.40	71.55	30.10	19.61	69.30	71.66
	Standing	64.10	62.47	27.00	20.76	61.20	64.12
	Kicking	54.30	72.67	38.30	80.47	59.80	84.28
	Average	65.22	78.34	45.98	63.65	68.33	82.07
**Hand Complex Movement**	Dribbling	72.30	90.96	74.80	91.75	80.30	93.43
	Catch	69.10	90.66	71.30	92.46	75.40	94.37
	Typing	81.20	77.22	51.20	82.84	84.20	84.69
	Writing	79.60	54.76	47.60	58.64	79.10	81.67
	Clapping	83.40	93.09	73.90	95.37	85.30	95.99
	Teeth	70.00	90.37	56.30	92.65	76.10	95.31
	Folding	60.00	79.54	38.80	74.17	63.00	76.12
	Pasta	67.20	82.39	38.10	81.90	71.60	82.81
	Soup	74.10	82.68	50.40	85.69	76.60	88.02
	Sandwich	61.90	74.28	37.60	53.65	62.10	78.08
	Chips	62.60	76.91	38.70	73.12	62.40	81.20
	Drinking	63.90	74.23	41.30	55.03	65.30	81.80
	Average	70.44	80.59	51.67	78.11	73.45	86.12

## Data Availability

Not applicable.

## Appendix A

**Table A1:** The summary of hyperparameters for the CNN network used in this work.

Stage	Hyperparameters		Values
Architecture	Convolution	Kernel Size	5
		Stride	1
		Filters	64
	Dropout		0.25
	Max Pooling		2
	Flatten		-
Training	Loss Function		Cross-entropy
	Optimizer		Adam
	Batch Size		64
	Number of Epochs		200

**Table A2:** The summary of hyperparameters for the LSTM network used in this work.

Stage	Hyperparameters	Values
Architecture	LSTM Unit	128
	Dropout	0.25
	Dense	128
Training	Loss Function	Cross-entropy
	Optimizer	Adam
	Batch Size	64
	Number of Epochs	200

**Table A3:** The summary of hyperparameters for the 1D-ResNet network used in this work.

Stage	Hyperparameters		Values
Architecture	**Convolutional Block**		
	Convolution	Kernel Size	5
		Stride	1
		Filters	64
	Batch Normalization		-
	Activation		ReLU
	Max Pooling		2
	**Residual Block × 8**		
	Convolution	Kernel Size	5
		Stride	1
		Filters	32
	Batch Normalization		-
	Activation		ReLU
	Convolution	Kernel Size	5
		Stride	1
		Filters	64
	Batch Normalization		-
	Global Average Pooling		-
	Flatten		-
	Dense		128
Training	Loss Function		Cross-entropy
	Optimizer		Adam
	Batch Size		64
	Number of Epochs		200

**Table A4:** The summary of hyperparameters for the 1D-ResNet-SE network used in this work.

Stage	Hyperparameters		Values
Architecture	Convolutional Block		
	Convolution	Kernel Size	5
		Stride	1
		Filters	64
	Batch Normalization		-
	Activation		ELU
	Max Pooling		2
	SE-ResNet Block × 8		
	Convolution	Kernel Size	5
		Stride	1
		Filters	32
	Batch Normalization		-
	Activation		ELU
	Convolution	Kernel Size	5
		Stride	1
		Filters	64
	Batch Normalization		-
	SE Module		-
	Global Average Pooling		-
	Flatten		-
	Dense		128
Training	Loss Function		Cross-entropy
	Optimizer		Adam
	Batch Size		64
	Number of Epochs		200

## References

[1] Ometov A., Shubina V., Klus L., Skibińska J., Saafi S., Pascacio P., Flueratoru L., Gaibor D.Q., Chukhno N., Chukhno O. (2021). A Survey on Wearable Technology: History, State-of-the-Art and Current Challenges. Comput. Netw..

[2] Nickel C., Wirtl T., Busch C. Authentication of Smartphone Users Based on the Way They Walk Using k-NN Algorithm. Proceedings of the 2012 Eighth International Conference on Intelligent Information Hiding and Multimedia Signal Processing.

[3] Lin C.L., Hwang T. (2003). A password authentication scheme with secure password updating. Comput. Secur..

[4] Merkle R.C., Pomerance C. (1988). A Digital Signature Based on a Conventional Encryption Function. Advances in Cryptology–CRYPTO ’87.

[5] Suh G.E., Devadas S. Physical Unclonable Functions for Device Authentication and Secret Key Generation. Proceedings of the 2007 44th ACM/IEEE Design Automation Conference.

[6] Indu I., Anand P.R., Bhaskar V. (2018). Identity and access management in cloud environment: Mechanisms and challenges. Eng. Sci. Technol. Int. J..

[7] Ailisto H., Lindholm M., Mäntyjärvi J., Vildjiounaite E., Mäkelä S.M. Identifying people from gait pattern with accelerometers. Proceedings of the Biometric Technology for Human Identification II.

[8] Derawi M.O., Nickel C., Bours P., Busch C. Unobtrusive User-Authentication on Mobile Phones Using Biometric Gait Recognition. Proceedings of the 2010 Sixth International Conference on Intelligent Information Hiding and Multimedia Signal Processing.

[9] Sha K., Kumari M. (2018). Patient Identification Based on Wrist Activity Data. Proceedings of the 2018 IEEE/ACM International Conference on Connected Health: Applications, Systems and Engineering Technologies.

[10] Wang C., Wang Y., Chen Y., Liu H., Liu J. (2020). User authentication on mobile devices: Approaches, threats and trends. Comput. Netw..

[11] Zhang S., Sun L., Mao X., Hu C., Liu P., Herrera L.J. (2021). Review on EEG-Based Authentication Technology. Comput. Intell. Neurosci..

[12] Burge M.J., Bowyer K.W. (2013). Handbook of Iris Recognition.

[13] Kavitha S., Sripriya P. (2018). A Review on Palm Vein Biometrics. Int. J. Eng. Technol..

[14] Saevanee H., Clarke N., Furnell S., Biscione V. (2015). Continuous user authentication using multi-modal biometrics. Comput. Secur..

[15] Kumar R., Phoha V.V., Raina R. (2016). Authenticating users through their arm movement patterns. arXiv.

[16] Chen L., Hoey J., Nugent C.D., Cook D.J., Yu Z. (2012). Sensor-Based Activity Recognition. IEEE Trans. Syst. Man Cybern. Part C (Appl. Rev.).

[17] Weiss G.M., Yoneda K., Hayajneh T. (2019). Smartphone and Smartwatch-Based Biometrics Using Activities of Daily Living. IEEE Access.

[18] Ahmad M., Alqarni M., Khan A., Khan A., Chauhdary S., Mazzara M., Umer T., Distefano S. (2018). Smartwatch-Based Legitimate User Identification for Cloud-Based Secure Services. Mob. Inf. Syst..

[19] Angrisano A., Bernardi M.L., Cimitile M., Gaglione S., Vultaggio M. (2020). Identification of Walker Identity Using Smartphone Sensors: An Experiment Using Ensemble Learning. IEEE Access.

[20] Mekruksavanich S., Jitpattanakul A. (2021). Biometric User Identification Based on Human Activity Recognition Using Wearable Sensors: An Experiment Using Deep Learning Models. Electronics.

[21] Benegui C., Ionescu R.T. (2020). Convolutional Neural Networks for User Identification Based on Motion Sensors Represented as Images. IEEE Access.

[22] Neverova N., Wolf C., Lacey G., Fridman L., Chandra D., Barbello B., Taylor G. (2016). Learning Human Identity From Motion Patterns. IEEE Access.

[23] He K., Sun J. Convolutional neural networks at constrained time cost. Proceedings of the 2015 IEEE Conference on Computer Vision and Pattern Recognition (CVPR).

[24] He K., Zhang X., Ren S., Sun J. Deep Residual Learning for Image Recognition. Proceedings of the 2016 IEEE Conference on Computer Vision and Pattern Recognition (CVPR).

[25] Li Y., Wang L. (2022). Human Activity Recognition Based on Residual Network and BiLSTM. Sensors.

[26] Ronald M., Poulose A., Han D.S. (2021). iSPLInception: An Inception-ResNet Deep Learning Architecture for Human Activity Recognition. IEEE Access.

[27] Hu J., Shen L., Sun G. Squeeze-and-Excitation Networks. Proceedings of the 2018 IEEE/CVF Conference on Computer Vision and Pattern Recognition.

[28] Mekruksavanich S., Jantawong P., Jitpattanakul A. A Lightweight Deep Convolutional Neural Network with Squeeze-and-Excitation Modules for Efficient Human Activity Recognition using Smartphone Sensors. Proceedings of the 2021 2nd International Conference on Big Data Analytics and Practices (IBDAP).

[29] Mekruksavanich S., Jitpattanakul A. Detection of Freezing of Gait in Parkinson’s Disease by Squeeze-and-Excitation Convolutional Neural Network with Wearable Sensors. Proceedings of the 2021 15th International Conference on Open Source Systems and Technologies (ICOSST).

[30] Mekruksavanich S., Jantawong P., Charoenphol A., Jitpattanakul A. Fall Detection from Smart Wearable Sensors using Deep Convolutional Neural Network with Squeeze-and-Excitation Module. Proceedings of the 2021 25th International Computer Science and Engineering Conference (ICSEC).

[31] Zou L., Liu W., Lei M., Yu X. (2021). An Improved Residual Network for Pork Freshness Detection Using Near-Infrared Spectroscopy. Entropy.

[32] Park J., Kim J.k., Jung S., Gil Y., Choi J.I., Son H.S. (2020). ECG-Signal Multi-Classification Model Based on Squeeze-and-Excitation Residual Neural Networks. Appl. Sci..

[33] Saini B.s., Kaur N., Bhatia K. (2018). Authenticating Mobile Phone User using Keystroke Dynamics. Int. J. Comput. Sci. Eng..

[34] Shi W., Yang J., Jiang Y., Yang F., Xiong Y. SenGuard: Passive user identification on smartphones using multiple sensors. Proceedings of the IEEE 7th International Conference on Wireless and Mobile Computing, Networking and Communications (WiMob).

[35] De Luca A., Hang A., Brudy F., Lindner C., Hussmann H. (2012). Touch Me Once and i Know It’s You! Implicit Authentication Based on Touch Screen Patterns. Proceedings of the SIGCHI Conference on Human Factors in Computing Systems.

[36] Sae-Bae N., Memon N., Isbister K. Investigating multi-touch gestures as a novel biometric modality. Proceedings of the 2012 IEEE Fifth International Conference on Biometrics: Theory, Applications and Systems (BTAS).

[37] Frank M., Biedert R., Ma E., Martinovic I., Song D. (2013). Touchalytics: On the Applicability of Touchscreen Input as a Behavioral Biometric for Continuous Authentication. IEEE Trans. Inf. Forensics Secur..

[38] Rocha C.C., Lima J.C.D., Dantas M.A.R., Augustin I. A2BeST: An adaptive authentication service based on mobile user’s behavior and spatio-temporal context. Proceedings of the 2011 IEEE Symposium on Computers and Communications (ISCC).

[39] Sabharwal M. (2017). Multi-Modal Biometric Authentication and Secure Transaction Operation Framework for E-Banking. Int. J. Bus. Data Commun. Netw..

[40] Jakobsson M., Shi E., Golle P., Chow R. (2009). Implicit Authentication for Mobile Devices. Proceedings of the 4th USENIX Conference on Hot Topics in Security.

[41] Casale P., Pujol O., Radeva P. (2012). Personalization and User Verification in Wearable Systems Using Biometric Walking Patterns. Pers. Ubiquitous Comput..

[42] Rong L., Jianzhong Z., Ming L., Xiangfeng H. A Wearable Acceleration Sensor System for Gait Recognition. Proceedings of the 2007 2nd IEEE Conference on Industrial Electronics and Applications.

[43] Mantyjarvi J., Lindholm M., Vildjiounaite E., Makela S.M., Ailisto H. Identifying users of portable devices from gait pattern with accelerometers. Proceedings of the IEEE International Conference on Acoustics, Speech, and Signal Processing.

[44] Parziale A., Carmona-Duarte C., Ferrer M.A., Marcelli A., Lladós J., Lopresti D., Uchida S. (2021). 2D vs 3D Online Writer Identification: A Comparative Study. Document Analysis and Recognition—ICDAR 2021.

[45] Musale P., Baek D., Werellagama N., Woo S.S., Choi B.J. (2019). You Walk, We Authenticate: Lightweight Seamless Authentication Based on Gait in Wearable IoT Systems. IEEE Access.

[46] Yang J.B., Nguyen M.N., San P.P., Li X.L., Krishnaswamy S. (2015). Deep Convolutional Neural Networks on Multichannel Time Series for Human Activity Recognition. Proceedings of the 24th International Conference on Artificial Intelligence.

[47] Ronao C.A., Cho S.B. (2016). Human activity recognition with smartphone sensors using deep learning neural networks. Expert Syst. Appl..

[48] Jiang W., Yin Z. (2015). Human Activity Recognition Using Wearable Sensors by Deep Convolutional Neural Networks. Proceedings of the 23rd ACM International Conference on Multimedia.

[49] Huang J., Lin S., Wang N., Dai G., Xie Y., Zhou J. (2020). TSE-CNN: A Two-Stage End-to-End CNN for Human Activity Recognition. IEEE J. Biomed. Health Inform..

[50] Ronneberger O., Fischer P., Brox T. (2015). U-Net: Convolutional Networks for Biomedical Image Segmentation. arXiv.

[51] Zhang Y., Zhang Z., Zhang Y., Bao J., Zhang Y., Deng H. (2019). Human Activity Recognition Based on Motion Sensor Using U-Net. IEEE Access.

[52] Mahmud T., Sazzad Sayyed A.Q.M., Fattah S.A., Kung S.Y. (2021). A Novel Multi-Stage Training Approach for Human Activity Recognition From Multimodal Wearable Sensor Data Using Deep Neural Network. IEEE Sens. J..

[53] Tang Y., Teng Q., Zhang L., Min F., He J. (2021). Layer-Wise Training Convolutional Neural Networks With Smaller Filters for Human Activity Recognition Using Wearable Sensors. IEEE Sens. J..

[54] Yang Z., Wang Y., Liu C., Chen H., Xu C., Shi B., Xu C., Xu C., Chaudhuri K., Salakhutdinov R. (2019). LegoNet: Efficient Convolutional Neural Networks with Lego Filters. Proceedings of the 36th International Conference on Machine Learning.

[55] Zeng M., Gao H., Yu T., Mengshoel O.J., Langseth H., Lane I., Liu X. (2018). Understanding and Improving Recurrent Networks for Human Activity Recognition by Continuous Attention. Proceedings of the 2018 ACM International Symposium on Wearable Computers.

[56] Barut O., Zhou L., Luo Y. (2020). Multitask LSTM Model for Human Activity Recognition and Intensity Estimation Using Wearable Sensor Data. IEEE Internet Things J..

[57] Aljarrah A.A., Ali A.H. Human Activity Recognition using PCA and BiLSTM Recurrent Neural Networks. Proceedings of the 2019 2nd International Conference on Engineering Technology and its Applications (IICETA).

[58] Steven Eyobu O., Han D.S. (2018). Feature Representation and Data Augmentation for Human Activity Classification Based on Wearable IMU Sensor Data Using a Deep LSTM Neural Network. Sensors.

[59] Chen Z., Jiang C., Xiang S., Ding J., Wu M., Li X. (2020). Smartphone Sensor-Based Human Activity Recognition Using Feature Fusion and Maximum Full A Posteriori. IEEE Trans. Instrum. Meas..

[60] Chen Z., Wu M., Gao K., Wu J., Ding J., Zeng Z., Li X. (2021). A Novel Ensemble Deep Learning Approach for Sleep-Wake Detection Using Heart Rate Variability and Acceleration. IEEE Trans. Emerg. Top. Comput. Intell..

[61] Cho K., van Merrienboer B., Gulcehre C., Bougares F., Schwenk H., Bengio Y. Learning phrase representations using RNN encoder-decoder for statistical machine translation. Proceedings of the Conference on Empirical Methods in Natural Language Processing (EMNLP 2014).

[62] Xu C., Chai D., He J., Zhang X., Duan S. (2019). InnoHAR: A Deep Neural Network for Complex Human Activity Recognition. IEEE Access.

[63] Chen Z., Wu M., Cui W., Liu C., Li X. (2021). An Attention Based CNN-LSTM Approach for Sleep-Wake Detection With Heterogeneous Sensors. IEEE J. Biomed. Health Inform..

[64] Chen K., Yao L., Zhang D., Wang X., Chang X., Nie F. (2020). A Semisupervised Recurrent Convolutional Attention Model for Human Activity Recognition. IEEE Trans. Neural Netw. Learn. Syst..

[65] Xia K., Huang J., Wang H. (2020). LSTM-CNN Architecture for Human Activity Recognition. IEEE Access.

[66] Alo U.R., Nweke H.F., Teh Y.W., Murtaza G. (2020). Smartphone Motion Sensor-Based Complex Human Activity Identification Using Deep Stacked Autoencoder Algorithm for Enhanced Smart Healthcare System. Sensors.

[67] Peng L., Chen L., Ye Z., Zhang Y. (2018). AROMA: A Deep Multi-Task Learning Based Simple and Complex Human Activity Recognition Method Using Wearable Sensors. Proc. ACM Interact. Mob. Wearable Ubiquitous Technol..

[68] Liu L., Peng Y., Liu M., Huang Z. (2015). Sensor-based human activity recognition system with a multilayered model using time series shapelets. Knowl.-Based Syst..

[69] Chen L., Liu X., Peng L., Wu M. (2021). Deep learning based multimodal complex human activity recognition using wearable devices. Appl. Intell..

[70] Shoaib M., Bosch S., Incel O.D., Scholten H., Havinga P.J.M. (2016). Complex Human Activity Recognition Using Smartphone and Wrist-Worn Motion Sensors. Sensors.

[71] Shoaib M., Scholten H., Havinga P.J.M., Incel O.D. A hierarchical lazy smoking detection algorithm using smartwatch sensors. Proceedings of the 2016 IEEE 18th International Conference on e-Health Networking, Applications and Services (Healthcom).

[72] Shoaib M., Incel O., Scholten H., Havinga P., Ohmura R., Murao K., Inoue S., Gotoh Y. (2018). Smokesense: Online activity recognition framework on smartwatches. Mobile Computing, Applications, and Services—9th International Conference, MobiCASE 2018.

[73] Harris E.J., Khoo I.H., Demircan E. (2022). A Survey of Human Gait-Based Artificial Intelligence Applications. Front. Robot. AI.

[74] Nguyen B., Coelho Y., Bastos T., Krishnan S. (2021). Trends in human activity recognition with focus on machine learning and power requirements. Mach. Learn. Appl..

[75] Scheurer S., Tedesco S., O’Flynn B., Brown K.N. (2020). Comparing Person-Specific and Independent Models on Subject-Dependent and Independent Human Activity Recognition Performance. Sensors.

[76] Reiss A., Stricker D. Introducing a New Benchmarked Dataset for Activity Monitoring. Proceedings of the 2012 16th International Symposium on Wearable Computers.

[77] Roggen D., Calatroni A., Rossi M., Holleczek T., Förster K., Tröster G., Lukowicz P., Bannach D., Pirkl G., Ferscha A. Collecting complex activity datasets in highly rich networked sensor environments. Proceedings of the 2010 Seventh International Conference on Networked Sensing Systems (INSS).

[78] Mostayed A., Kim S., Mazumder M.M.G., Park S.J. Foot Step Based Person Identification Using Histogram Similarity and Wavelet Decomposition. Proceedings of the 2008 International Conference on Information Security and Assurance (ISA 2008).

[79] Anguita D., Ghio A., Oneto L., Parra Perez X., Reyes Ortiz J.L. A Public Domain Dataset for Human Activity Recognition using Smartphones. Proceedings of the Networks, Computational Intelligence and Machine Learning (ESANN 2013).

[80] Kwapisz J.R., Weiss G.M., Moore S.A. (2011). Activity Recognition Using Cell Phone Accelerometers. SIGKDD Explor. Newsl..

[81] Hastie T., Tibshirani R., Friedman J. (2009). The Elements of Statistical Learning: Data Mining, Inference, and Prediction.

[82] Monti R.P., Tootoonian S., Cao R. (2018). Avoiding Degradation in Deep Feed-Forward Networks by Phasing out Skip-Connections.

[83] Muqeet A., Iqbal M.T.B., Bae S.H. (2019). HRAN: Hybrid Residual Attention Network for Single Image Super-Resolution. IEEE Access.

[84] Bisong E. (2019). Building Machine Learning and Deep Learning Models on Google Cloud Platform: A Comprehensive Guide for Beginners.

[85] Abadi M., Agarwal A., Barham P., Brevdo E., Chen Z., Citro C., Corrado G.S., Davis A., Dean J., Devin M. (2016). TensorFlow: Large-Scale Machine Learning on Heterogeneous Distributed Systems. arXiv.

[86] Ismail Fawaz H., Forestier G., Weber J., Idoumghar L., Muller P.A. (2019). Deep learning for time series classification: A review. Data Min. Knowl. Discov..

[87] Benavoli A., Corani G., Mangili F. (2016). Should We Really Use Post-Hoc Tests Based on Mean-Ranks?. J. Mach. Learn. Res..

[88] Hodges J.L., Lehmann E.L. (1962). Rank Methods for Combination of Independent Experiments in Analysis of Variance. Ann. Math. Stat..

[89] Finner H. (1993). On a Monotonicity Problem in Step-Down Multiple Test Procedures. J. Am. Stat. Assoc..

